# Supplementary material for: Genetic Variation in CYP2D6, UGT1A4, SLC6A2 and SLCO1B1 Alters the Pharmacokinetics and Safety of Mirabegron
Source: Pharmaceutics. 2024 Aug 17;16(8):1077. doi: 10.3390/pharmaceutics16081077 (PMC11359404; doi:10.3390/pharmaceutics16081077)
Supplement: Supplementary file 1 [file pharmaceutics-16-01077-s001.zip › pharmaceutics-3145479-supplementary.pdf]

Genetic Variation in *CYP2D6, UGT1A4, SLC6A2* and *SLCO1B1* Alters the Pharmacokinetics and Safety of Mirabegron

Paula Soria-Chacartegui [1], Patricia Cendoya-Ramiro [1], Eva González-Iglesias [1], Samuel Martín-Vílchez [1], Andrea Rodríguez-Lopez [1], Gina Mejía-Abril [1], Manuel Román [1], Sergio Luquero-Bueno [1], Dolores Ochoa [1] and Francisco Abad-Santos [1,2,*]

[1] Clinical Pharmacology Department, Hospital Universitario de La Princesa, Faculty of Medicine, Instituto de Investigación Sanitaria La Princesa (IP), Universidad Autónoma de Madrid (UAM), 28006 Madrid, Spain
[2] Centro de Investigación Biomédica en Red de Enfermedades Hepáticas y Digestivas (CIBERehd), Instituto de Salud Carlos III, 28029 Madrid, Spain
* Correspondence: francisco.abad@uam.es

SUPPLEMENTARY MATERIAL

Supplementary Table S1. Analyzed genetic variants.

Gene	SNV	Nucleotide change[a]	Allele(s) containing the SNV
ABCB1	rs1045642	T>C	NA
	rs1128503	T>C	NA
	rs2032582	T>G	NA
		T>A	NA
ABCC2	rs2273697	G>A	NA
	rs3740066	C>T	NA
ABCC3	rs4793665	C>T	NA
ABCG2	rs2231142	C>A	NA
CES1	rs2244613	C>A	NA
	rs71647871	G>A	NA
	rs8192935	T>C	NA
CYP1A2	rs12720461	C>T	NA
	rs2069514	G>A	NA
	rs2069526	T>G	NA
	rs2470890	T>C	NA
	rs72547516	A>T	NA
	rs762551	C>A	NA
CYP2A6	rs28399433	A>C	NA
CYP2B6	rs2279343	A>G	*4, *6, *7, *18, *36
	rs28399499	T>C	*18
	rs3211371	C>T	*5, *7
	rs34223104	T>C	*22, *36
	rs3745274	G>T	*6, *7, *9, *36
CYP2C18	rs11188059	G>A	NA
	rs2860840	C>T	NA

CYP2C19	rs12248560	C>T	*17
	rs12769205	A>G	*2, *35
	rs17884712	G>A	*9
	rs28399504	A>G	*4
	rs41291556	T>C	*8
	rs4244285	G>A	*2
	rs4986893	G>A	*3
	rs56337013	C>T	*5
	rs72552267	G>A	*6
	rs72558186	T>C	*7
CYP2C8	rs10509681	A>G	*3
	rs1058930	C>G	*4
	rs11572080	G>A	*3
	rs11572103	A>T	*2
CYP2C9	rs1057910	A>C	*3
	rs1799853	C>T	*2
	rs28371685	C>T	*11
	rs28371686	C>G	*5
	rs7900194	G>A	*8
CYP2D6	rs1065852	C>T	*1, *4, *10; *49, *56, *64, *65, *69, *114
	rs1135822	T>A	*49
	rs1135840	G>C	*2, *4, *6, *8, *10, *12, *14, *17, *19, *29, *31, *39, *41, *42, *49, *56, *59, *64, *65, *69, *114
	rs16947	C>T	*2, *4, *8, *12, *14, *17, *19, *29, *31, *34, *41, *42, *56, *59, *65, *69, *114
	rs267608319	G>A	*31
	rs28371706	C>T	*17, *64
	rs28371725	G>A	*41, *69, *119
	rs35742686	A>delA	*3
	rs3892097	G>A	*4
	rs5030655	T>delT	*6
	rs5030656	GAA>delGAA	*9, *109
	rs5030862	G>A	*12
	rs5030865	G>T	*8, *14, *114
	rs5030867	A>C	*7
	rs59421388	G>A	*29, *109
	rs61736512	G>A	*29, *107
	rs72549346	GTG>GTGTG	*42
	rs72549347	C>T	*56
	rs72549353	AACT>CT	*19
	rs774671100	T>TT	*15
	rs79292917	G>A	*59
CYP3A4	rs2242480	G>A	*36
	rs2740574	G>A	*1A, *1B
	rs28371759	T>C	*18
	rs35599367	C>T	*22, *37
	rs4646438	A>AA	*6
	rs4986910	T>C	*3, *37
	rs55785340	T>C	*2
	rs55901263	C>G	*5
	rs55951658	A>G	*4

	rs67666821	T>TTT	*20
CYP3A43	rs61469810	A>delA	NA
CYP3A5	rs10264272	G>A	*6
	rs41303343	T>TT	*7
	rs776746	A>G	*3
CYP4F2	rs114099324	G>C	*7
	rs2108622	G>A	*3, *4
	rs3093105	T>G	*2, *4
	rs3093153	G>T	*6
	rs3093200	C>A	*5
EPHX1	rs1051740	T>C	NA
	rs2234922	A>G	NA
NAT2	rs1799930	G>A	*6
	rs1799931	G>A	*7
	rs1801280	T>C	*5
SLC19A1	rs1051266	A>G	NA
SLC22A1	rs12208357	C>T	NA
	rs34059508	G>A	NA
	rs628031	A>G	NA
	rs72552763	GAT>delGAT	NA
SLC22A2	rs316019	T>G	NA
SLC28A3	rs7853758	C>T	NA
SLC6A2	rs12708954	C>A	NA
	rs3785143	C>T	NA
SLCO1B1	rs11045819	C>A	*4, *14
	rs2306283	A>G	*14, *15, *20, *31, *37
	rs34671512	A>C	*19, *20, *40
	rs373327528	G>A	*23
	rs4149056	T>C	*5, *15
	rs59502379	G>C	*9, *31
UGT1A	rs10929302	G>A	NA
UGTA1	rs4148323	G>A	*6
	rs887829	C>T	*80
UGT1A3	rs2008584	A>G	NA
UGT1A4	rs2011425	T>G	NA
UGT1A6	rs10445704	G>A	NA
	rs7592281	G>T	NA
UGT1A8	rs1042597	C>G	NA
UGT2B10	rs61750900	G>T	NA
UGT2B15	rs1902023	A>C	NA
UGT2B7	rs7668258	T>C	NA

[a]: *Nucleotide variants are annotated according to reference sequences provided by the National Center for Biotechnology Information (NCBI) Reference Sequence (RefSeq) database.*

Supplementary Table S2. No statistically significant associations between genetic variation and pharmacokinetic parameters.

Gene	Genotype/ Diplotype/ Phenotype		n	AUC/ DW kg·ng·h/mL·mg	C_{max}/DW kg·ng/mL·mg	t_{max} h	$t_{1/2}$ h	CL/F L/kg·h
ABCB1	rs1045642	T/T	14	525.01 (179.50)	48.49 (23.24-65.64)	4.19 (3.84-4.78)	27.93 (3.80)	1.92 (1.63-2.67)
		T/C	36	583.34 (198.07)	52.93 (31.93-72.18)	4.39 (3.75-5.00)	25.45 (6.22)	1.78 (1.36-2.49)
		C/C	29	531.72 (180.73)	43.15 (24.90-73.46)	4.88 (4.00-5.81)	26.90 (8.65)	1.94 (1.57-2.61)
	rs1128503	T/T	13	491.86 (159.54)	48.18 (21.59-64.55)	4.13 (3.69-4.81)	28.62 (4.76)	2.19 (1.67-2.91)
		T/C	35	583.63 (195.59)	57.72 (33.97-74.89)	4.50 (3.75-5.13)	24.70 (5.32)	1.81 (1.32-2.38)
		C/C	31	546.74 (178.10)	48.74 (28.87-73.27)	4.88 (4.00-5.75)	27.45 (8.71)	1.92 (1.53-2.51)
	rs2032582	T/T	9	465.27 (153.88)	48.18 (22.86-55.84)	4.13 (3.75-4.50)	28.67 (4.04)	2.19 (1.71-3.17)
		T/A	3	694.31 (204.59)	82.62 (65.11-85.51)	4.38 (4.07-4.94)	20.80 (3.25)	1.32 (1.31-2.75)
		G/A	6	540.05 (143.65)	60.74 (13.81-7.94)	5.06 (4.22-7.81)	28.08 (3.51)	1.78 (1.53-2.56)
		G/T	27	569.83 (195.43)	53.99 (29.50-72.57)	4.50 (3.63-5.13)	25.38 (5.98)	1.81 (1.42-2.61)
		G/G	29	553.85 (185.25)	48.74 (32.20-73.57)	4.50 (4.00-5.31)	27.25 (8.49)	1.92 (1.56-2.61)
ABCC2	rs2273697	G/G	60	548.61 (184.12)	51.30 (26.58-73.09)	4.50 (4.00-5.25)	26.18 (7.18)	1.92 (1.55-2.52)
		G/A+A/A	19	571.26 (187.71)	48.61 (34.86-70.71)	4.38 (3.63-5.00)	27.20 (6.00)	1.87 (1.47-2.53)
	rs3740066	C/C	31	530.48 (173.79)	48.74 (28.59-63.47)	4.38 (3.63-5.00)	27.65 (8.68)	1.87 (1.66-2.74)
		C/T	38	556.78 (194.00)	48.57 (28.66-72.74)	4.50 (4.00-5.31)	26.26 (5.39)	1.98 (1.50-2.55)
		T/T	10	616.77 (178.36)	72.49 (44.83-89.72)	4.63 (3.72-5.22)	23.24 (5.02)	1.63 (1.29-2.24)
ABCC3	rs4793665	C/C	6	582.80 (178.71)	48.67 (34.65-71.65)	4.25 (3.34-4.50)	29.60 (7.25)	1.94 (1.54-2.15)
		C/T	45	543.38 (63.91)	52.97 (32.15-70.37)	4.41 (3.81-5.13)	24.81 (4.93)	1.91 (1.63-2.41)
		T/T	28	565.05 (218.28)	45.85 (19.64-74.58)	4.81 (4.00-5.75)	28.45 (8.82)	1.83 (1.31-2.77)
ABCG2	rs2231142	C/C	56	570.16 (189.32)	53.48 (33.49-73.82)	4.44 (3.79-4.97)	25.86 (6.90)	1.87 (1.43-2.48)
		C/A	18	530.98 (162.74)	48.17 (28.18-63.63)	4.45 (3.69-5.69)	27.51 (6.75)	1.97 (1.70-2.54)
		A/A	3	393.17 (207.89)	19.98 (19.81-41.73)	5.13 (4.63-5.25)	23.89 (1.28)	3.55 (2.58-3.73)
CES1	rs2244613	C/C	3	451.42 (175.69)	19.02 (16.45-41.25)	4.88 (4.51-7.94)	29.66 (3.72)	2.28 (1.95-2.92)
		C/A	22	583.71 (197.75)	62.88 (32.48-85.38)	4.50 (4.00-5.28)	26.09 (8.58)	1.81 (1.29-2.59)
		A/A	53	549.98 (180.30)	48.44 (31.01-70.37)	4.38 (3.75-5.06)	26.35 (6.35)	1.91 (1.57-2.51)
	rs71647871	G/G	76	548.23 (181.98)	49.73 (29.87-71.01)	4.50 (3.79-5.13)	26.35 (6.87)	1.92 (1.54-2.53)
		G/A	1	661.50	73.66	4.88	24.37	1.53
	rs8192935	T/T	6	518.92 (182.71)	39.93 (16.91-73.36)	4.50 (3.19-6.41)	27.44 (7.60)	2.01 (1.54-2.94)
		T/C	44	557.71 (177.47)	54.75 (35.27-73.82)	4.45 (3.91-5.22)	25.58 (6.91)	1.84 (1.47-2.50)
		C/C	27	558.39 (204.92)	42.22 (28.87-67.23)	4.50 (3.75-5.13)	27.83 (6.79)	1.92 (1.51-2.52)

CYP1A2	rs12720461	C/C	77	556.76 (185.02)	50.72 (30.23-71.82)	4.50 (3.825.13)	26.52 (6.94)	1.87 (1.52-2.51)
		C/T	1	544.25	75.89	4.75	19.48	1.94
	rs2069514	G/G	47	529.21 (191.61)	40.91 (28.02-69.25)	4.63 (4.00-5.38)	27.31 (7.42)	2.01 (1.62-2.78)
		G/A	18	623.71 (184.75)	61.99 (44.85-87.31)	4.39 (3.56-5.03)	25.24 (5.25)	1.71 (1.32-1.99)
		A/A	4	494.88 (108.59)	48.17 (27.20-66.61)	4.31 (3.88-5.78)	25.50 (10.18)	1.99 (1.73-2.66)
	rs2069526	T/T	70	565.11 (183.68)	49.73 (31.04-72.55)	4.50 (3.85-5.13)	26.46 (7.04)	1.87 (1.50-2.44)
		T/G+G/G	9	468.10 (172.71)	53.99 (17.45-67.16)	4.50 (3.88-6.19)	26.14 (6.01)	2.25 (1.81-3.44)
	rs2470890	T/T	11	640.83 (205.60)	51.87 (28.87-88.40)	4.50 (4.00-6.50)	27.46 (6.46)	1.47 (1.31-2.21)
		T/C	38	528.17 (178.07)	45.33 (30.59-67.14)	4.50 (3.85-5.13)	27.37 (7.61)	1.95 (1.64-2.64)
		C/C	28	562.90 (180.25)	60.14 (29.52-73.56)	4.39 (3.66-5.19)	24.67 (6.13)	1.81 (1.52-2.41)
	rs72547516	A/A	77	554.23 (186.35)	50.72 (30.23-91.52)	4.50 (3.82-5.23)	26.37 (6.79)	1.91 (1.52-2.52)
	rs762551	C/C	8	554.90 (229.02)	61.26 (24.72-99.21)	4.69 (3.88-5.25)	24.09 (4.92)	2.17 (1.33-3.00)
		C/A	33	530.22 (178.44)	42.22 (31.01-66.93)	4.75 (3.69-5.13)	27.71 (8.34)	1.98 (1.58-2.66)
		A/A	37	574.35 (184.16)	55.51 (28.73-74.38)	4.38 (3.94-5.13)	25.99 (5.64)	1.81 (1.46-2.24)
CYP2A6	rs28399433	A/A	58	567.24 (188.25)	51.30 (32.77-70.81)	4.50 (3.85-5.03)	26.33 (5.81)	1.84 (1.46-2.51)
		A/C	13	559.66 (182.26)	53.99 (28.45-90.53)	4.41 (3.69-5.63)	27.34 (11.65)	2.01 (1.50-2.49)
		C/C	4	417.93 (138.26)	32.63 (15.74-58.14)	4.06 (3.44-6.09)	27.45 (4.52)	2.76 (1.83-3.36)
CYP2B6	Phenotype	RM	3	585.86 (263.42)	51.87 (33.29-71.42)	6.50 (5.38-6.88)	23.08 (3.04)	1.45 (1.39-2.53)
		NM	31	514.00 (186.42)	42.22 (20.93-65.95)	4.50 (3.76-5.38)	26.71 (6.16)	2.21 (1.67-2.82)
		IM	35	565.75 (179.40)	50.72 (34.00-73.27)	4.50 (3.75-5.13)	26.60 (7.91)	1.92 (1.47-2.38)
		PM	5	643.68 (161.24)	52.97 (48.46-82.22)	4.25 (4.06-4.33)	28.37 (7.01)	1.74 (1.39-1.84)
CYP2C18	rs11188059	G/G	47	573.99 (182.99)	55.50 (31.06-71.11)	4.50 (4.00-5.38)	27.32 (8.11)	1.81 (1.47-2.41)
		G/A	18	530.82 (171.69)	44.91 (26.92-70.94)	4.63 (3.72-5.13)	25.01 (4.88)	2.05 (1.63-2.65)
		A/A	2	311.19 (4.19)	25.22 [20.93-29.50]	5.00 [3.75-6.25]	27.53 (1.63)	3.28 [3.25-3.32]
	rs2860840	C/C	23	568.21 (180.72)	48.74 (34.00-70.71)	4.50 (4.00-7.88)	27.80 (6.83)	1.91 (1.64-2.38)
		C/T	26	500.97 (178.40)	36.98 (22.81-62.12)	4.94 (3.94-6.06)	27.01 (8.08)	2.24 (1.75-2.89)
		T/T	12	623.66 (179.88)	65.35 (38.11-98.50)	4.19 (3.63-5.22)	25.41 (7.33)	1.57 (1.37-1.89)
CYP2C19	Phenotype	UM	2	463.19 (193.70)	31.51 [22.11-40.91]	4.56 [4.13-5.00]	28.97 (1.47)	2.46 [1.66-3.26]
		RM	11	578.94 (213.72)	63.14 (34.86-82.62)	4.50 (3.75-4.88)	24.20 (5.24)	1.69 (1.32-2.53)
		NM	33	544.81 (197.94)	48.61 (23.51-79.53)	4.50 (3.69-5.56)	27.01 (8.68)	1.92 (1.48-2.77)
		IM	19	537.34 (144.76)	48.18 (33.33-63.30)	4.25 (3.76-5.00)	26.92 (5.88)	1.93 (1.74-2.41)
CYP2C8	Phenotype	UM+RM	10	588.49 (170.00)	60.14 (32.69-93.77)	4.13 (3.56-5.53)	25.08 (6.41)	1.79 (1.42-2.11)
		NM	61	558.26 (191.91)	51.87 (30.23-71.63)	4.50 (3.88-5.25)	26.68 (7.01)	1.92 (1.49-2.57)
		IM	5	469.68 (130.40)	42.22 (20.35-59.65)	4.75 (4.13-5.94)	24.48 (4.36)	2.41 (1.69-2.98)

		UND	2	580.67 (13.36)	60.65 [48.74-72.57]	4.00 [3.75-4.25]	28.47 (15.81)	1.75 [1.70-1.80]
CYP2C9	Phenotype	NM	56	558.25 (190.01)	49.73 (33.98-71.01)	4.50 (4.00-5.22)	26.39 (6.97)	1.92 (1.48-2.48)
		IM	18	563.92 (170.07)	60.74 (28.80-72.74)	4.38 (3.63-4.91)	26.07 (6.60)	1.81 (1.63-2.64)
CYP3A4	Phenotype	NM	74	553.71 (189.25)	49.73 (29.34-70.81)	4.50 (3.76-5.16)	26.65 (6.98)	1.92 (1.50-2.55)
		IM	2	593.71 (15.11)	57.09 [40.91-73.27]	4.50 [4.00-5.00]	23.24 (6.63)	1.72 [1.66-1.81]
CYP3A43	rs61469810	A/A	61	563.69 (185.22)	51.87 (32.20-72.55)	4.38 (3.75-5.19)	26.32 (7.09)	1.87 (1.52-2.41)
		A/delA+delA/delA	16	532.35 (187.79)	54.97 (28.76-73.19)	4.63 (4.06-5.06)	26.09 (6.03)	1.92 (1.43-3.04)
CYP3A5	Phenotype	NM+IM	57	562.98 (167.96)	54.24 (44.85-72.54)	4.19 (3.85-4.91)	26.24 (6.19)	1.80 (1.62-2.16)
		PM	18	545.29 (191.32)	47.60 (28.73-70.57)	4.75 (3.82-5.25)	26.78 (7.14)	1.94 (1.52-2.66)
CYP4F2	Diplotype	*1/*1	25	529.07 (132.58)	48.54 (29.83-64.71)	4.25 (3.81-5.19)	27.51 (9.13)	1.91 (1.68-2.41)
		*1/*X	23	516.38 (178.17)	48.61 (28.02-70.03)	4.88 (3.75-5.25)	26.33 (5.23)	1.97 (1.66-2.82)
		*X/*X	10	588.73 (222.91)	36.92 (23.30-86.60)	5.13 (4.41-6.81)	27.02 (7.73)	1.94 (1.29-2.74)
EPHX1	rs1051740	T/T	35	541.05 (196.74)	48.54 (28.02-65.95)	4.75 (4.00-5.25)	27.05 (5.77)	1.98 (1.61-2.61)
		T/C	36	569.73 (178.54)	53.69 (34.21-73.09)	4.39 (4.78-5.00)	25.93 (7.83)	1.81 (1.47-2.40)
		C/C	5	531.67 (178.25)	59.2 (22.21-86.26)	4.00 (3.56-5.94)	25.80 (8.91)	1.92 (1.54-2.88)
	rs2234922	A/A	46	582.33 (189.89)	58.36 (29.34-63.88)	4.50 (3.75-5.03)	26.35 (7.83)	1.84 (1.44-2.43)
		A/G+G/G	30	519.59 (175.39)	48.36 (29.34-63.88)	4.45 (3.94-5.41)	26.60 (5.48)	1.92 (1.66-2.84)
NAT2	Diplotype	*1/*1	7	602.63 (159.64)	60.84 (48.74-65.11)	4.00 (3.26-4.25)	27.80 (7.77)	1.75 (1.68-1.93)
		*1/*5	22	599.44 (200.55)	61.46 (31.67-85.98)	4.38 (3.76-5.28)	25.03 (4.36)	1.64 (1.34-2.37)
		*1/*6	5	486.14 (188.60)	36.51 (26.82-87.79)	3.75 (3.31-5.19)	22.37 (6.16)	2.38 (1.56-3.13)
		*1/*7	7	631.26 (191.40)	63.30 (48.61-73.66)	4.38 (4.00-4.88)	24.88 (6.28)	1.87 (1.22-2.18)
		*5/*5	7	560.90 (214.45)	43.15 (21.07-74.89)	4.25 (3.50-6.75)	25.83 (3.88)	2.26 (1.26-2.41)
		*6/*6	2	456.31 (209.42)	35.21 [29.50-40.91]	4.38 [3.75-5.00]	28.31 (0.53)	2.45 [1.66-3.25]
		*5/*6	16	500.97 (135.49)	38.09 (24.86-64.86)	4.75 (4.13-5.06)	27.64 (6.98)	2.10 (1.78-2.68)
		*5/*7	6	462.65 (248.08)	28.58 (19.40-45.65)	5.31 (4.84-5.78)	30.60 (6.26)	2.97 (1.46-3.74)
		*6/*7	5	584.23 (168.07)	70.71 (29.71-80.31)	4.25 (3.69-5.81)	27.96 (17.45)	1.69 (1.48-2.56)
		*7/*7	1	552.15	55.50	4.41	23.32	2.09
NUDT15	Phenotype	NM	74	546.04 (181.45)	49.67 (29.87-70.54)	4.50 (3.88-5.13)	26.51 (6.80)	1.92 (1.61-2.52)
		IM	2	731.14 (126.41)	93.71 [48.74-138.68]	4.25 [3.38-5.12]	25.67 (12.54)	1.31 [1.22-1.40]
SLC22A1	rs12208357	C/C	76	550.83 (184.90)	51.30 (29.03-72.18)	4.45 (3.79-5.22)	26.42 (7.01)	1.92 (1.52-2.53)
		C/T	3	635.84 (168.34)	48.44 (44.16-89.16)	4.88 (4.69-4.94)	26.57 (3.53)	1.87 (1.57- 2.03)
	rs34059508	G/G	76	560.67 (184.01)	52.42 (29.87-72.56)	4.50 (3.88-5.22)	26.39 (7.00)	1.87 (1.51-2.52)

		G/A	2	357.69 (98.37)	26.82 [19.63-34.00]	4.19 [3.25-5.13]	26.88 (5.24)	2.96 [2.38-3.55]
	rs628031	A/A	8	508.32 (189.73)	45.20 (21.65-72.92)	4.63 (4.16-5.16)	29.80 (12.27)	2.08 (1.57-3.19)
		A/G	30	531.09 (167.40)	42.03 (27.97-72.74)	4.50 (4.00-5.03)	26.52 (6.00)	1.92 (1.66-2.54)
		G/G	40	577.57 (196.73)	53.69 (33.72-71.71)	4.39 (3.66-5.34)	25.57 (6.14)	1.81 (1.33-2.48)
	rs72552763	GAT/GAT	47	548.52 (183.38)	48.44 (30.96-73.66)	4.50 (3.88-5.00)	25.99 (7.39)	1.87 (1.53-2.61)
		GAT/delGAT	24	554.89 (183.33)	49.67 (28.66-65.74)	4.63 (4.00-5.44)	27.61 (6.03)	1.92 (1.38-2.48)
		delGAT/delGAT	7	613.61 (209.88)	63.30 (60.84-72.54)	4.13 (3.26-6.25)	23.77 (5.27)	1.68 (1.22-2.41)
SLC22A2	rs316019	T/G	20	591.72 (167.07)	60.74 (42.06-82.20)	4.39 (4.00-4.97)	25.34 (9.09)	1.81 (1.50-198)
		G/G	59	541.29 (189.07)	48.18 (28.02-70.03)	4.50 (3.75-5.25)	26.79 (6.03)	1.94 (1.52-2.61)
SLC28A3	rs7853758	C/C	44	582.12 (187.85)	60.14 (36.03-75.39)	4.31 (3.75-4.88)	24.94 (6.73)	1.77 (1.44-2.20)
		C/T	30	539.10 (173.75)	40.44 (30.22-65.32)	4.63 (3.84-5.56)	27.47 (4.32)	2.03 (1.61-2.64)
		T/T	3	458.03 (166.23)	26.10 (19.99-44.79)	5.13 (4.63-8.07)	28.02 (3.31)	2.28 (1.95-2.86)
SLC28A3	rs3785143	C/C	61	539.15 (183.36)	48.61 (29.19-71.84)	4.50 (3.94-5.13)	26.55 (7.50)	1.93 (1.52-2.57)
		C/T	18	604.55 (182.30)	60.14 (33.15-73.37)	4.45 (3.63-5.28)	25.98 (4.39)	1.74 (1.33-2.13)
SLCO1B1	Phenotype	NF	53	568.51 (187.45)	57.72 (33.67-72.92)	4.38 (3.81-5.06)	25.67 (5.82)	1.81 (1.46-2.52)
		DF	23	536.17 (177.76)	36.51 (23.61-63.30)	4.50 (4.00-5.88)	28.29 (8.99)	1.97 (1.66-2.41)
TPMT	Phenotype	NM	63	560.10 (186.78)	51.87 (29.50-70.03)	4.50 (3.88-5.25)	26.95 (7.28)	1.91 (1.51-2.51)
		IM	12	521.52 (147.03)	44.72 (35.47-73.55)	4.25 (3.78-5.00)	24.72 (5.18)	1.90 (1.67-2.49)
UGT1A	rs10929302	G/G	35	554.32 (177.74)	52.97 (30.96-69.25)	4.38 (3.75-5.00)	26.57 (6.42)	1.81 (1.53-2.52)
		G/A	36	563.48 (194.98)	49.73 (29.03-74.31)	4.50 (4.00-5.47)	26.62 (7.86)	1.89 (1.36-2.52)
		A/A	7	505.68 (190.35)	34.86 (19.63-73.88)	5.13 (3.75-6.25)	25.69 (3.73)	2.07 (1.62-3.39)
UGT1A1	Phenotype	NM	27	579.80 (183.88)	55.50 (31.06-72.54)	4.38 (3.63-5.00)	26.13 (6.65)	1.75 (1.51-2.41)
		IM	43	542.69 (183.52)	48.74 (28.59-71.11)	4.63 (4.00-5.38)	27.03 (7.50)	1.93 (1.52-2.53)
		PM	8	494.66 (178.97)	36.16 (23.22-65.40)	4.50 (3.56-5.97)	25.24 (3.68)	2.14 (1.69-3.20)
UGT1A6	rs10445704	G/G	23	565.17 (162.05)	52.97 (31.06-70.03)	4.25 (3.75-4.88)	27.65 (7.62)	1.81 (1.53-2.41)
		G/A	44	550.51 (198.34)	50.24 (26.72-74.63)	4.50 (3.91-5.47)	26.33 (6.76)	1.92 (1.43-2.66)
		A/A	10	572.47 (180.94)	44.09 (32.69-76.07)	4.69 (3.97-5.25)	25.00 (6.34)	2.00 (1.41-2.31)
	rs7592281	G/G	68	556.46 (182.79)	49.73 (30.99-72.18)	4.45 (3.79-5.09)	26.43 (5.99)	1.92 (1.51-2.48)
		G/T	5	655.83 (198.93)	73.88 (30.14-84.31)	5.00 (3.69-5.88)	23.42 (3.97)	1.62 (1.27-2.35)
UGT1A8	rs1042597	C/C	44	537.37 (192.12)	47.89 (26.58-69.84)	4.50 (3.75-5.22)	26.43 (7.34)	2.04 (1.55-2.71)
		C/G	29	576.40 (165.25)	53.99 (38.19-70.91)	4.25 (3.94-5.13)	26.86 (6.63)	1.81 (1.56-2.26)
		G/G	3	563.48 (197.61)	53.99 (52.31-82.32)	4.50 (4.38-4.69)	22.48 (2.53)	1.53 (1.49-2.33)
UGT2B10	rs61750900	G/G	69	552.50 (183.79)	48.74 (29.19-72.55)	4.50 (3.94-5.19)	26.75 (7.03)	1.91 (1.52-2.52)
		G/T	8	534.06 (160.35)	49.96 (29.35-72.63)	4.13 (3.66-5.22)	24.30 (4.95)	2.00 (1.67-2.71)

		T/T	2	687.58 (354.62)	66.27 [63.30-69.25]	3.94 [3.38-4.50]	23.61 (10.59)	1.75 [1.09-2.41]
UGT2B15	rs1902023	A/A	10	590.32 (201.34)	57.28 (38.06-77.34)	4.44 (3.76-4.88)	27.39 (4.88)	1.66 (1.41-2.45)
		A/C	45	542.14 (188.53)	50.72 (27.06-70.25)	4.50 (3.81-5.19)	26.22 (7.35)	1.92 (1.56-2.76)
		C/C	24	561.28 (173.47)	53.99 (34.63-72.18)	4.25 (3.91-5.44)	26.39 (6.94)	1.81 (1.54-2.49)
UGT2B7	rs7668258	T/T	10	584.97 (191.81)	58.13 (42.92-75.13)	4.19 (3.94-4.50)	25.30 (5.93)	2.07 (1.29-2.44)
		T/C	25	531.30 (188.16)	48.61 (24.85-72.19)	4.88 (4.39-5.25)	26.53 (8.19)	1.92 (1.52-3.10)
		C/C	41	568.58 (184.01)	51.87 (29.19-72.55)	4.25 (3.75-5.13)	26.75 (6.53)	1.81 (1.49-2.39)

Results are shown as mean (standard deviation) for normally-distributed variables and median (interquartile range) for not normally-distributed variables. For categories with two volunteers, the two values in square brackets are shown instead of the interquartile range. AUC/DW: dose/weight corrected area under the time-concentration curve; C_{max} /DW: dose/weight maximum drug plasma concentration; t_{max}: time to reach C_{max}; $t_{1/2}$: elimination half-life; UM: ultrarapid metabolizers; RM: rapid metabolizers; NM: normal metabolizers; IM: intermediate metabolizers; PM: poor metabolizers; IF: increased function; NF: normal function; DF: decreased function; PF: poor function; del: deletion, UND: undetermined.
